# Strain effects on the electronic properties of cobalt-based coordination nanosheets

**DOI:** 10.1039/d5na00385g

**Published:** 2025-09-01

**Authors:** Kento Nishigomi, Yu Yi, Souren Adhikary, Kazuhito Tsukagoshi, Katsunori Wakabayashi

**Affiliations:** a Department of Nanotechnology for Sustainable Energy, School of Science and Technology, Kwansei Gakuin University Gakuen-Uegahara 1 Sanda 669-1330 Japan waka@kwansei.ac.jp +81 79 565 9729 +81 79 565 9751; b Research Center for Materials Nanoarchitectonics (MANA), National Institute for Materials Science (NIMS) Namiki 1-1 Tsukuba 305-0044 Japan; c Center for Spintronics Research Network (CSRN), Osaka University Toyonaka 560-8531 Japan

## Abstract

We theoretically study strain effects on the electronic properties of cobalt-based benzenehexathiol (CoBHT) coordination nanosheets using first-principles calculations. Two distinct crystal structures, high-density structure (HDS) and low-density structure (LDS), are explored. Our results reveal that HDS behaves as a metal, while LDS exhibits semiconducting properties. Spin-polarized electronic band structures highlight the presence of energy band structures of the Kagome lattice, and the inclusion of spin–orbit coupling (SOC) results in band gap openings at high-symmetric *K* points. Furthermore, we construct a tight-binding model to investigate the topological properties of CoBHT, demonstrating anomalous Hall conductivity driven by the intrinsic Berry curvature. The impact of uniaxial strain on the electronic and magnetic properties of CoBHT is also studied. Strain induces significant modifications in magnetic moments and density of states, particularly in the HDS. Anomalous Hall conductivity is enhanced under hole-doping conditions, suggesting that strain can be used to tailor the electronic properties of CoBHT for specific applications. Our findings underscore the potential of CoBHT nanosheets for use in next-generation electronic, optoelectronic, and catalytic devices with tunable properties through strain engineering.

## Abstract 2

We theoretically study strain effects on the electronic properties of cobalt-based benzenehexathiol (CoBHT) coordination nanosheets using first-principles calculations.

## Graphical abstract

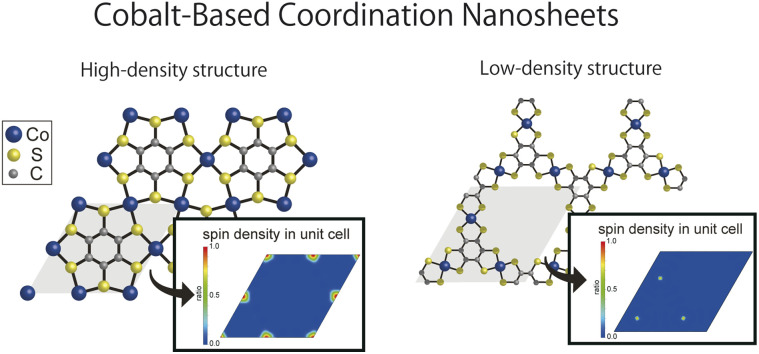

## 1 Introduction

Two-dimensional (2D) materials, such as graphene,^1–4^ boron-nitrides,^5,6^ transition metal dichalcogenides (TMDCs),^7–9^ and oxide nanosheets,^10^ have garnered significant attention due to their unique physical and chemical properties, *i.e.*, spin and charge transport,^11–14^ ferroelectricity,^15–17^ magnetism,^18,19^ and layer-by-layer oxidation.^20,21^ Moreover, the nontrivial topological properties of 2D materials give rise to edge and corner states and optical shift currents, offering promising applications in electronic, spintronic, and quantum devices.^22–29^ These materials, often obtained *via* top-down exfoliation from bulk-layered crystals, exhibit unique physical properties driven by their reduced dimensionality. However, bottom-up approaches, where nanosheets are synthesized through molecular, ionic, or atomic bonds, offer a complementary method to tailor 2D material properties and create novel structures. Coordination nanosheets (CONASHs),^30–33^ a class of 2D materials composed of metal–organic frameworks, represent one such bottom-up approach, enabling the design of nanosheets with versatile electronic, magnetic, and optical characteristics.

The material properties of CONASHs can be fine-tuned by selecting different metal centers and ligands, covering a broad range of the periodic table. The incorporation of transition metals into these materials especially enhances their functionality. Notable examples include the interfacial synthesis of semiconducting nickel bis(dithiolene) (NiBHT) nanosheets,^32,34^ photo-functional bis(dipyrrinato)zinc nanosheets,^35^ and electrochromic iron or cobalt bis(terpyridine) nanosheets.^36^ Recent studies by Clough *et al.* further revealed the potential of transition-metal dithiolene complex coordination polymers in hydrogen evolution catalysis.^37^ Theoretical work by Liu *et al.* has predicted that single-layer NiBHT could function as a 2D topological insulator.^38^

In this paper, using density functional theory we study electronic properties of cobalt-based coordination nanosheets (CoBHT),^39^ constructed from cobalt, sulfur, and carbon atoms. Since CoBHT is a 2D thin film, applying uniaxial strain is considered to significantly modify the electronic properties owing to the mechanical flexibility, similar to that observed in TMDCs, *i.e.*, so-called strain engineering.^40–45^ Thus, in this paper, we study the effect of external strain on the electronic properties of CoBHT. As previously reported, other transition metal-based BHT compounds have been experimentally synthesized in two crystal phases: high-density structure (HDS) and low-density structure (LDS), *via* a liquid–liquid interfacial reaction. CuBHT and FeBHT have been realized in the HDS phase,^46–48^ whereas NiBHT has been realized in the LDS phase.^32^ Based on these experimental observations, we investigate the possibility that CoBHT could exist in both HDS and LDS phases, as both exhibit a *D*_6h_ symmetry with a periodic arrangement of dithiolene groups. Clough *et al.* have reported Fourier-transform infrared (FTIR) spectra of CoBHT in the LDS phase^37^ and Li *et al.* theoretically reported the HDS phase.^49^ However, the precise crystal structure of CoBHT has not yet been experimentally confirmed. Here we explore the electronic and topological properties of these two different crystal structures using first-principles calculations to identify the suitable crystal structure. Additionally, we investigate how external strain influences the electronic and magnetic behavior of CoBHT, offering insights into the tunability of these properties for practical applications.

In Sec. II, we present the details of the crystal structure of CoBHT, and study the electronic states for both HDS and LDS using first-principles calculations. It will be shown that HDS becomes a spin-polarized metallic state. However, LDS becomes a spin-polarized semiconductor which is similar to NiBHT.^38^ Energetically LDS is slightly more stable than HDS. Since Co atoms form the Kagome lattice structure, the energy band structures have Dirac cones and flat bands. It is also pointed out that the spin–orbit interactions cause a small opening of the energy band gap at Dirac cones. In Sec. III, we deduce an effective tight-binding model by constructing maximally localized Wannier functions^50,51^ for CoBHT. Since HDS has local magnetic moments originating from Co atoms, it shows an anomalous Hall effect (AHE) owing to the finite Berry curvature. In Sec. IV, we study the external strain effect on the electronic states of CoBHT. The DOS for HDS near the Fermi energy is shown to be sensitive to the external strain; however, there is almost no change for LDS due to its semiconducting nature. In Sec. V, we provide the summary of the paper. In the SI, we provide strain effects of CoBHT on electronic band structures beyond 5% and a simple tight-binding analysis of the charge density profile of CoBHT.

## 2 Electronic states of CoBHT

**Fig. 1:**
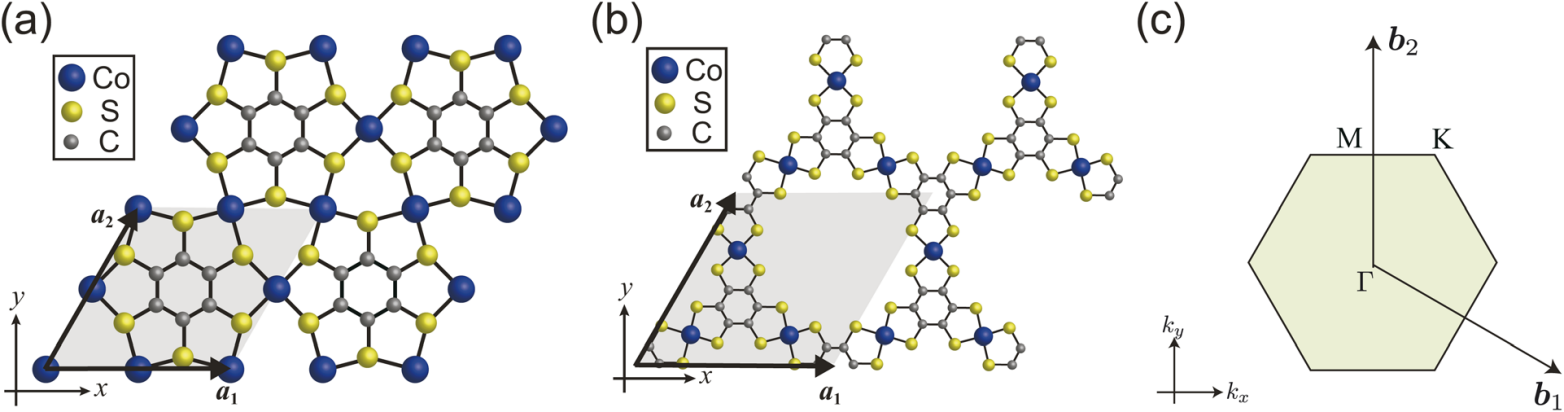
Crystal structures of CoBHT: (a) high-density structure (HDS) and (b) low-density structure (LDS). The gray rhombus is the unit cell. CoBHT consists of Co (blue), S (yellow), and C (gray) atoms. Here, ***a***_1_ = (*a*, 0) and 
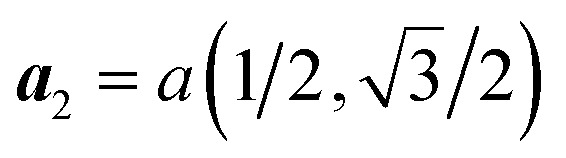
 are primitive vectors, where *a* is the lattice constant. For HDS and LDS, *a* = 8.45 and 14.52 Å, respectively. (c) The corresponding 1st Brillouin zone (BZ). Here, 
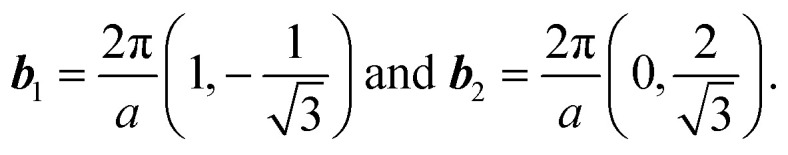

CoBHT is a newly synthesized coordination nanosheet composed of cobalt, sulfur, and carbon atoms. Two crystal structures have been proposed for CoBHT, as shown in Fig. 1(a) and (b): HDS and LDS, respectively. Both structures exhibit a periodic arrangement of dithiolene groups and possess *D*_6h_ symmetry. It should be noted that the Co atoms form a Kagome lattice structure in both cases. The gray region represents the unit cell, and the primitive lattice vectors for both structures are *a*_1_ = (1, 0)*a* and 
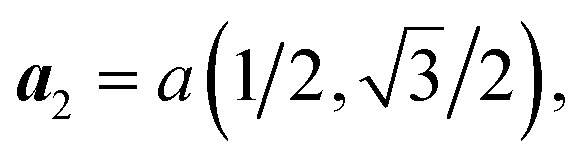
 with lattice constants of *a* = 8.45 Å for HDS and *a* = 14.52 Å for LDS. The corresponding primitive vectors in reciprocal space are given as 
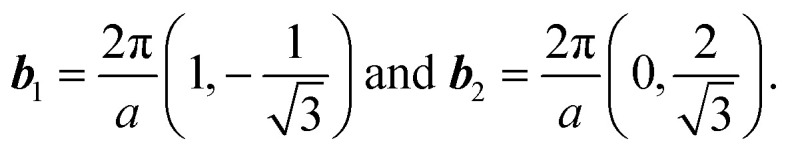
 Therefore, the corresponding first Brillouin zone (BZ) is shown in Fig. 1(c).

In this paper, electronic structure calculations were performed with a density functional theory (DFT)-based Quantum Espresso package using the projector augmented wave pseudopotential method.^52^ The exchange correlation function was considered using the generalized gradient approximation (GGA) by the Perdew–Burke–Ernzerhof (PBE) method.^53^ We performed structural optimization of CoBHT using a variable cell relaxation procedure. For structural optimization, the kinetic energy cut off was set to 85 Ry and the Brillouin zone was sampled using a 12 × 12 × 1 grid based on a *Γ*-centered Monkhorst–Pack mesh.^54^ The convergence threshold for the forces on each atom was set below 10^−5^ Ry Å^−1^, and the convergence criterion for the energy was set to 10^−13^ Ry.

**Fig. 2:**
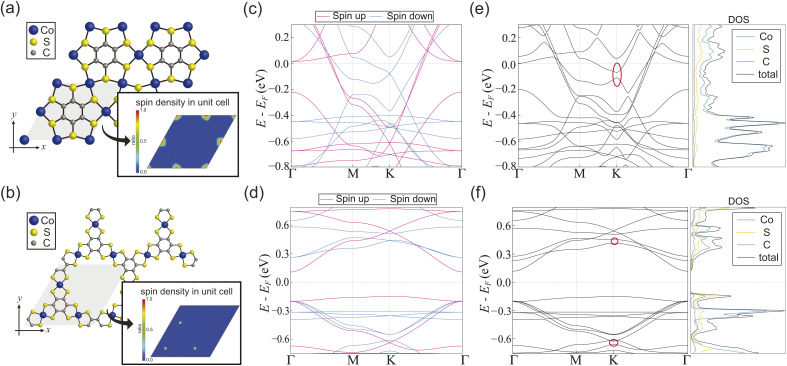
Spin density plots of CoBHT in the unit cell for (a) HDS and (b) LDS, respectively. CoBHT has finite magnetic moments originating from Co atoms. The magnetic moment of HDS is 1.85 μ_B_, while that of LDS is 4.07 μ_B_. Spin-polarized electronic band structures for (c) HDS and (d) LDS, respectively. Red and blue lines represent up and down spin states, respectively. Figures (e) and (f) depict the electronic band structures and density of states (DOS) considering SOC for HDS and LDS, respectively. The band gap openings at the *K* point are marked by red ellipses. In the DOS plots, the blue lines represent the cobalt atoms, yellow lines represent sulfur atoms, gray lines represent carbon atoms, and black lines represent the total DOS. LDS exhibits semiconductor behavior with a band gap of 0.265 eV.

Fig. 2(a) and (b) show the spin density plots within the unit cell for HDS and LDS, respectively. Both HDS and LDS exhibit finite magnetic moments, which originate from the cobalt atoms. The ground state of both systems exhibits ferromagnetic ordering. To verify this, we have compared the total energies of the systems in a ferromagnetic configuration and an antiferromagnetic configuration (in which one of the Co atoms is aligned oppositely to the other two). We have found that the ferromagnetic configuration has a lower total energy. The magnitudes of these magnetic moments are 1.85 μ_B_ for HDS and 4.07 μ_B_ for LDS. The difference in the magnitude of magnetic moments between the HDS and LDS phases can be attributed to the role of the ligand, *i.e.*, the different structural connectivity of Co atoms in the two phases (see Fig. 1(a) and (b)). To confirm this, we calculate the Bader charges on each Co atom in both systems.^55^ We find that the average Bader charge on a Co atom is 8.29 (in arbitrary unit) in the HDS system and 8.25 (in arbitrary unit) in the LDS system. As a result, the magnitude of the magnetic moment differs between the two systems. It is also worth comparing the binding energy, Δ*E*, between HDS and LDS. The binding energy is calculated using the following equation:1
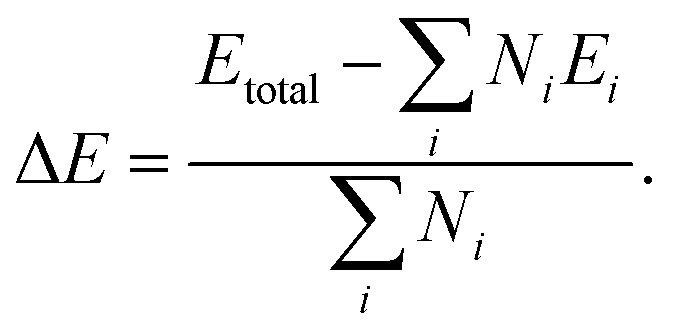
Here, the summation index *i* represents the Co, C and S atoms. *E*_total_ is the total energy of CoBHT for either HDS or LDS. *N*_*i*_ and *E*_*i*_ denote the total number and total energy of the *i* atom, respectively. The calculated values of Δ*E* are −0.5165 Ry per atom for LDS and −0.5065 Ry per atom for HDS, indicating that both HDS and LDS are energetically stable. Moreover, LDS is slightly more stable than HDS by 0.0010 Ry per atom. Further, we evaluated the thermodynamic stability using molecular dynamics simulations at 300 K, with the results presented in the SI.^56^ We observed that both phases of CoBHT remained intact, preserving their hexagonal configurations and planarity compared to the 0 K structures. These findings confirm the thermal stability of both phases.

Fig. 2(c) and (d) show the spin-polarized electronic energy band structure for HDS and LDS, respectively. The red and blue lines indicate the spin-up and spin-down states, respectively. The electronic energy band structure is calculated along highly symmetric directions in the first BZ. HDS is metallic. However, LDS becomes a semiconductor with a band gap of 0.265 eV. Both structures exhibit linear dispersion at the *K* point and feature a band structure resembling the Kagome lattice (Kagome-like band). Additionally, we have calculated the electronic band structures by including an on-site Coulomb interaction (DFT + U) on each Co atom for both systems and present the results in the SI. We find no changes in their magnetic ground state and electronic band dispersion. This result is consistent with a previous theoretical study.^57^ In the SI, considering the simple tight-binding model of the Kagome lattice, we compare the charge density plots obtained from DFT with those obtained from the simple tight-binding model.

Since CoBHT contains cobalt atoms, which induce relatively large intrinsic spin–orbit interactions into the system, here we have taken spin–orbit coupling (SOC) into account. Fig. 2(e) and (f) depict the electronic energy band structures with SOC, together with the partial density of states (PDOS) for HDS and LDS, respectively. In both structures, finite SOC opens the energy band gap at the *K* point (as marked by the red ellipses). Furthermore, from the PDOS near the Fermi energy, it is evident that in HDS the contribution of cobalt electrons is predominant, while in the LDS, there is a contribution not only from cobalt electrons but also from sulfur and carbon electrons.

## 3 Tight-binding model and Berry curvature

In order to analyze the topological properties of CoBHT, we shall construct an effective tight-binding model of HDS using Wannier90 (ref. 58) to reproduce the energy band structure obtained by DFT. In HDS, the electronic states near the Fermi energy are predominantly occupied by cobalt electrons. Furthermore, since the d-orbitals of cobalt electrons contribute in this energy range, we consider all d orbitals (d_*xy*_, d_*xz*_, d_*yz*_, d_*z*^2^_, d_*x*^2^−*y*^2^_) for each of the three cobalt atoms in the unit cell, *i.e.*, a total of 15 orbitals. The size of the effective tight-binding Hamiltonian is 30 × 30, because the SOC derived from cobalt atoms is also considered.

**Fig. 3:**
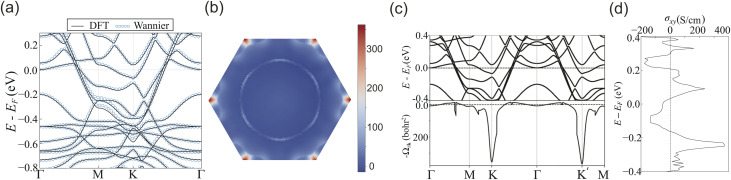
(a) Comparison of the band structures of CoBHT for HDS with SOC as calculated by DFT (the black line) and the WTB Hamiltonian (the blue circles). The WTB Hamiltonian consists of all d-orbitals of the cobalt atoms. (b) Contour plot of Berry curvature (−*Ω*_*z****k***_) in the first BZ. (c) Energy band structure and corresponding Berry curvature (−*Ω*_*z****k***_) along the path through the high-symmetric points in the first BZ. (d) Fermi energy dependence of the anomalous Hall conductivity of CoBHT for HDS.

Fig. 3(a) shows the energy band obtained by DFT and the effective model for HDS obtained by Wannier90. The black lines represent the energy band dispersion from DFT, while the blue circles represent the energy band dispersion from the effective tight-binding model. Thus, the energy band structure of HDS of CoBHT can be well-described by the d orbitals of cobalt atoms. Since the cobalt atoms form a 2D katome lattice, further analysis using a simple tight-binding model is presented in the SI.

Since HDS has a spin-polarized metallic state owing to the local magnetic moment of Co atoms, it is expected that HDS exhibits AHE,^59–65^*i.e.*, finite Hall conductivity without an external magnetic field. It is known that there are two main mechanisms for AHE, *i.e.*, extrinsic and intrinsic mechanisms. The extrinsic one is attributed to the skew scattering^63,64^ or side-jump^65^ from disorder. The intrinsic one can be attributed to the topological properties of bulk wave functions, which occur even in the perfect crystal. Here, we shall focus on the intrinsic AHE. The anomalous Hall conductivity can be obtained by the ***k***-integration of the Berry curvature in the 1st BZ as2
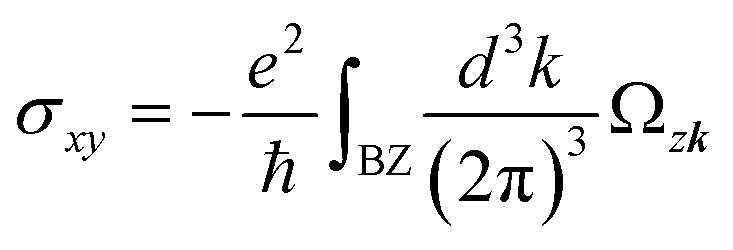
Here *Ω*_*z****k***_ is the summation of Berry curvature up to the Fermi energy, which is given as3
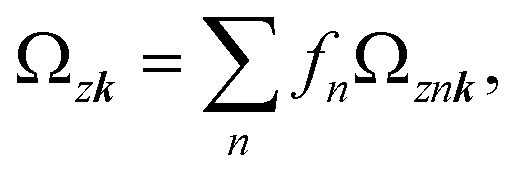
where *f*_*n*_ is the Fermi-Dirac distribution function and *n* is the band index. The Berry curvature for the *n*-th band can be numerically evaluated through the Kubo formula, *i.e.*,4
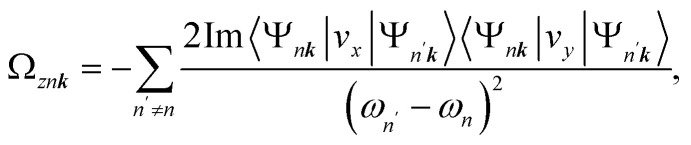
where *v*_*x*(*y*)_ is the *x*(*y*) component of the velocity operators, *ω*_*n*_ = *E*_*n*_/*ℏ*.

Since CoBHT is ferromagnetic, *i.e.*, a time-reversal broken system, the Berry curvature has the property of *Ω*_*z*,***k***_ = *Ω*_*z*,−***k***_. Fig. 3(b) shows the contour plot of Berry curvature *Ω*_*z*,***k***_ in the 1st BZ. Fig. 3(c) shows the energy band structure of HDS near the Fermi energy and the corresponding Berry curvature *Ω*_*z*,***k***_ along the high symmetric ***k*** points of the 1st BZ. The Berry curvature of HDS clearly exhibits a six-fold rotational symmetry with respect to the *Γ* point. It is clearly seen that the most pronounced peaks appear at *K* and *K*′ points, where Dirac points exist owing to the nature of the 2D Kagome lattice. Thus, as shown in Fig. 3(d), the finite anomalous Hall conductivity *σ*_*xy*_ is obtained for CoBHT, which is a profound value.

## 4 Strain effect on CoBHT

In atomically-thin 2D materials, the electronic states can be significantly modified by the application of external strain.^66–68^ Here we study the strain effect on the electronic states of CoBHT. Since CoBHT has *D*_6h_ hexagonal symmetry, the application of uniaxial strain breaks the hexagonal crystal symmetry, resulting in the significant modification of electronic states. Furthermore, recent studies show that the strain can induce topological transitions in the 2D Kagome lattice.^69^

**Fig. 4:**
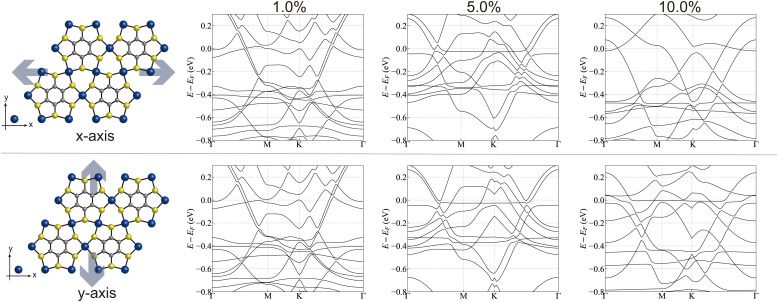
Strain effect of CoBHT for HDS on the electronic energy band structures. (Upper panels) Elongation strain along the *x*-axis with strain values of 1.0, 5.0, and 10.0%. (Lower panels) Elongation strain along the *y*-axis.

Fig. 4 shows the energy band structures of HDS under the application of external strains of 1.0%, 5.0%, and 10.0%, respectively. Here, the elongation strain is applied. The effect of compression strain and weaker strain less than 1.0% is presented in the SI. The upper and lower panels of Fig. 4 correspond to the external strain along the *x* and *y* axes, respectively. Since applying the strain to HDS breaks the hexagonal symmetry of the system, a gap opens up in the linear dispersion at the *K* point.

It is also observed that strain induces an anisotropic effect in the electronic states of CoBHT, which becomes sizable in the range of 5.0% to 10.0%. However, the following discussion shall focus on the uniaxial strain up to 0.5%, from an experimental perspective. In the case of biaxial strain, further large strain can be achieved using indenting devices.^70–72^

**Fig. 5:**
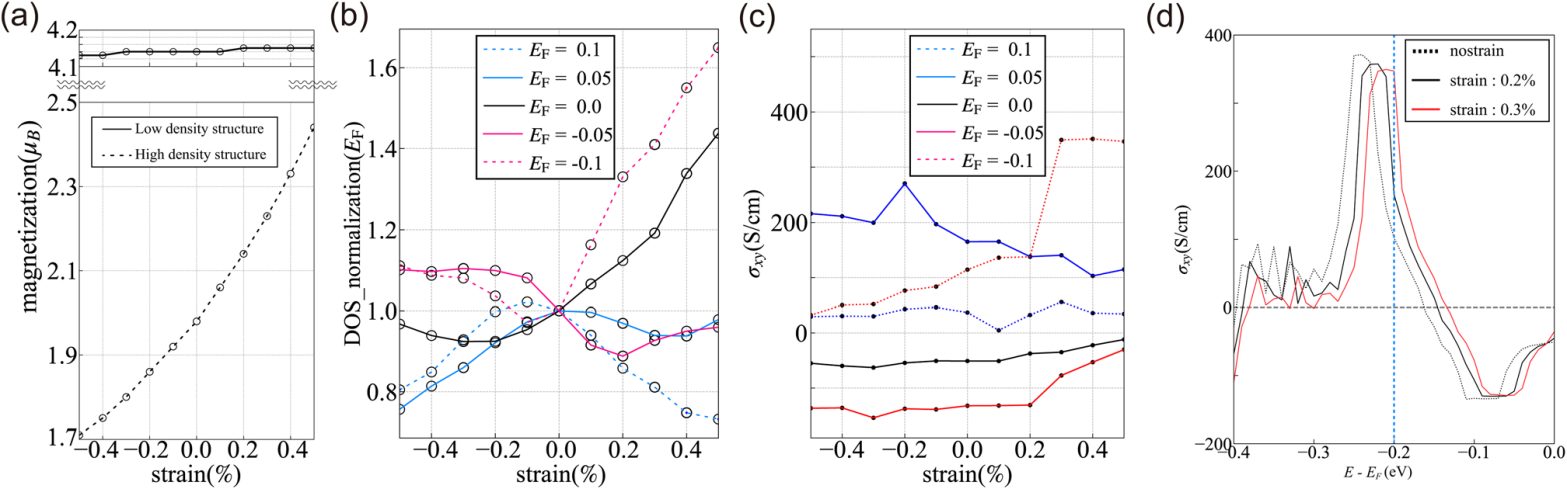
(a) Strain dependence of magnetic moments for (lower) HDS and (upper) LDS, respectively. Here *E*_F_ is fixed at 0 eV, *i.e.*, non-doping case. (b) The strain effect of DOS at *E*_F_ for HDS is for several different electron or hole doping cases. (c) Strain effect on the anomalous Hall conductivity of HDS. (d) Fermi energy dependence of anomalous Hall conductivity for HDS with several different strains.

Fig. 5(a) illustrates the strain dependence of the magnetic moment for HDS and LDS of CoBHT at the charge-neutral point, *i.e.*, *E*_F_ = 0. For HDS, the magnetic moment monotonically increases with an increase in strain. However, for LDS, the magnetic moment remains nearly unchanged. Thus, the HDS has a stronger strain dependence of magnetic moments than the LDS. Since the magnetic moments originate from the d-orbitals of Co atoms, these results indicate that the strain affects the spin–spin interactions in HDS more than LDS. This might be attributed to the fact that the atomic distances between Co atoms differ significantly between HDS and LDS. In other words, HDS (LDS) has stronger (weaker) magnetic interactions between Co atoms.

Fig. 5(b) shows the strain dependence of DOS for HDS at several different Fermi energies. The values of DOS are normalized by the DOS at *E*_F_ = 0 with no strain. At the charge neutral point, applying a 0.5% strain results in an approximately 40% increase in the DOS compared to the case without strain. Taking the doped case into consideration, it was found that applying a 0.5% strain, especially in the hole-doped case (*E*_F_ = −0.1 eV), results in an approximately 60% increase. One anticipated application of CONASHs is their utilization as electrode catalyst nanosheets, and the results suggest the possibility of activating catalytic functions. The LDS has a band gap of 0.265 eV, so there is no DOS in the range where *E*_F_ is from −0.1 to 0.1.

Fig. 5(c) depicts the anomalous Hall conductivity of HDS obtained by applying strain and calculating it at several different Fermi energy levels, similar to the procedure employed for DOS calculations. The calculation of anomalous Hall conductivity was performed using eqn (2) presented in Sect. III. The change in conductivity due to strain is generally small on average, but for the hole-doped case with *E*_F_ = −0.2, a significant increase was observed when applying strain from 0.2% to 0.3%.

Fig. 5(d) shows graphs for cases where the strain of 0.2% and 0.3% was applied, with the horizontal axis representing Fermi energy and the vertical axis indicating conductivity. The blue dashed line shows *E*_F_ = −0.2. Upon comparing each graph, it can be observed that when applying strain from 0.2% to 0.3%, the peaks near the blue dashed line precisely overlap. Therefore, when strain is applied at 0.3% or higher, the conductivity takes on significant values.

## 5 Conclusions

In this paper, we investigated the electronic structure and strain effects of CoBHT using first-principles calculations. Our results demonstrate that CoBHT exhibits diverse electronic and magnetic properties, with the HDS showing metallic behavior and the LDS functioning as a semiconductor with a band gap of 0.265 eV. The inclusion of SOC further reveals a band gap opening at the *K* points, contributing to the topological properties of the system. We also analyzed the Kagome-like band structure and anomalous Hall conductivity using the Wannier tight-binding model, confirming the non-trivial topological nature of the HDS.

Furthermore, we explored strain effects on the electronic properties of CoBHT, showing that uniaxial strain can induce significant changes in magnetic moments, DOS, and anomalous Hall conductivity. These findings suggest that strain engineering could be a viable approach to enhance the electronic functionality of CoBHT, particularly in applications requiring tunable electronic, magnetic, and catalytic properties. This work underscores the potential of CoBHT nanosheets for next-generation electronic and optoelectronic devices, as well as advanced catalytic applications.

## Author contributions

Kento Nishigomi: Formal analysis, investigation, data curation, writing – original draft, visualization, software. Yu Yi: Investigation. Souren Adhikary: Investigation, formal analysis. Kazuhito Tsukagoshi: Conceptualization, writing – review & editing. Katsunori Wakabayashi: Conceptualization, methodology, project administration, supervision, writing – original draft, writing – review & editing.

## Conflicts of interest

There are no conflicts to declare.

## Supplementary Material

NA-OLF-D5NA00385G-s001

## Data Availability

Additional data or computational files are available from the corresponding author upon reasonable request. All relevant data supporting the findings of this study are included in the article and its SI. See DOI: https://doi.org/10.1039/d5na00385g.
