# Supplementary material for: Strain effects on the electronic properties of cobalt-based coordination nanosheets
Source: Nanoscale Adv. 2025 Sep 1;7(21):6964–71. doi: 10.1039/d5na00385g (PMC12451603; doi:10.1039/d5na00385g)
Supplement: NA-007-D5NA00385G-s001 [file NA-007-D5NA00385G-s001.pdf]

Supplementary Information (SI) for Nanoscale Advances.
This journal is © The Royal Society of Chemistry 2025

Supplemental Material for "Strain Effects on Electronic Properties of Cobalt-Based Coordination Nanosheets"

Kento Nishigomi,[1] Yu Yi,[1] Souren Adhikary,[1] Kazuhito Tsukagoshi,[2] and Katsunori Wakabayashi[1,2,3]

[1] *Department of Nanotechnology for Sustainable Energy, School of Science and Technology, Kwansei Gakuin University, Gakuen-Uegahara 1, Sanda 669-1330, Japan*
[2] *Research Center for Materials Nanoarchitectonics (MANA), National Institute for Materials Science (NIMS), Namiki 1-1, Tsukuba 305-0044, Japan*
[3] *Center for Spintronics Research Network (CSRN), Osaka University, Toyonaka 560-8531, Japan*
(Dated: August 17, 2025)

In this Supplemental Material, we provide several supporting data for the main text. In Sec.I, we study the charge density profile of Kagome-like energy band obtained from DFT for CoBHT of high-density structure (HDS). It is shown that the charge density profiles can be understood by the simple tight-binding model of two-dimensional (2D) Kagome lattice. In Sec. II, we study the energy band structure of HDS under uniaxial strain of $0.0, 0.1, 0.2, 0.3, 0.5\%$. Both cases of elongation and compression strains are presented. In Sec. III, we study the anisotropic effect of strain in the regime of strong strain beyond 5.0%. In Sec. IV, we show the thermal stability of CoBHT in both HDS and LDS phase using molecular dynamics simulation at 300 K. In Sec. V, we study the electronic band structure of CoBHT using DFT+U method. We add on-site Coulomb potential (U in eV) on each Co atoms.

I. COMPARISON WITH TIGHT BINDING MODEL OF KAGOME LATTICE

In CoBHT, cobalt atoms form the two-dimensional kagome lattice. Here, we show that the energy band structure and wave functions obtained by DFT calculation can be well described by those obtained by a simple tight-binding model for the Kagome lattice. As an example, we shall analyze the energy band of the spin-up state for the HDS of CoBHT.

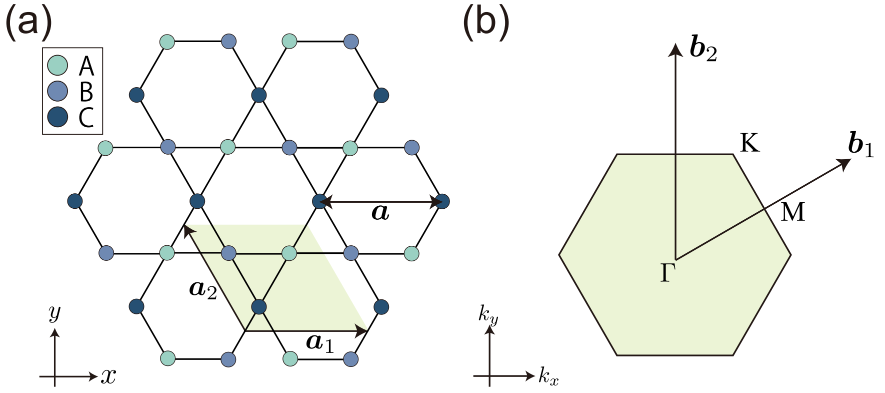

FIG. S1. (a) Schematic lattice structure of kagome lattice. (b) Corresponding 1st Brillouine Zone.

Figure S1(a) represents the crystal structure of the kagome lattice. The shaded rhombus denotes the unit cell, which contains three nonequivalent atomic sites called A, B and C. The primitive vectors are $\boldsymbol{a}_1 = (1,0)a$ and $\boldsymbol{a}_2 = (1/2, \sqrt{3}/2)a$, where a is the lattice constant. The corresponding reciprocal lattice vectors are $b_1 = \frac{2\pi}{a}(1, \frac{1}{\sqrt{3}})$ and $b_2 = \frac{2\pi}{a}(0, \frac{2}{\sqrt{3}})$, respectively. Thus, Fig. S1(b) shows the 1st Brillouin Zone (BZ) of the kagome lattice. The high symmetric points in 1st BZ are $\Gamma = (0,0)$, $\mathrm{K} = (\frac{4\pi}{3a}, 0)$ and $\mathrm{M} = (\pi, \frac{\pi}{\sqrt{3}a})$.

Here, we consider the tight-binding model with only nearest-neighbor electron hopping on the 2D kagome lattice. The eigenvalue equation can be written as

$$\mathcal{H}(\boldsymbol{k}) \left|\Psi_{n,\boldsymbol{k}}\right\rangle = E_{n,\boldsymbol{k}} \left|\Psi_{n,\boldsymbol{k}}\right\rangle . \tag{S1}$$

Here, $\mathcal{H}(\boldsymbol{k})$ is the Hamiltonian at the wavevector $\boldsymbol{k} = (k_x, k_y)$, and $E_{n,\boldsymbol{k}}$ is the eigenvalue with the band index $n(= 1, 2, 3)$. The wave function is $\left|\Psi_{n,\boldsymbol{k}}\right\rangle = [c_{n,A}(\boldsymbol{k}), c_{n,B}(\boldsymbol{k}), c_{n,C}(\boldsymbol{k})]^T$, where $c_{n,\alpha}(\boldsymbol{k})$ is the amplitude of the wavefunction

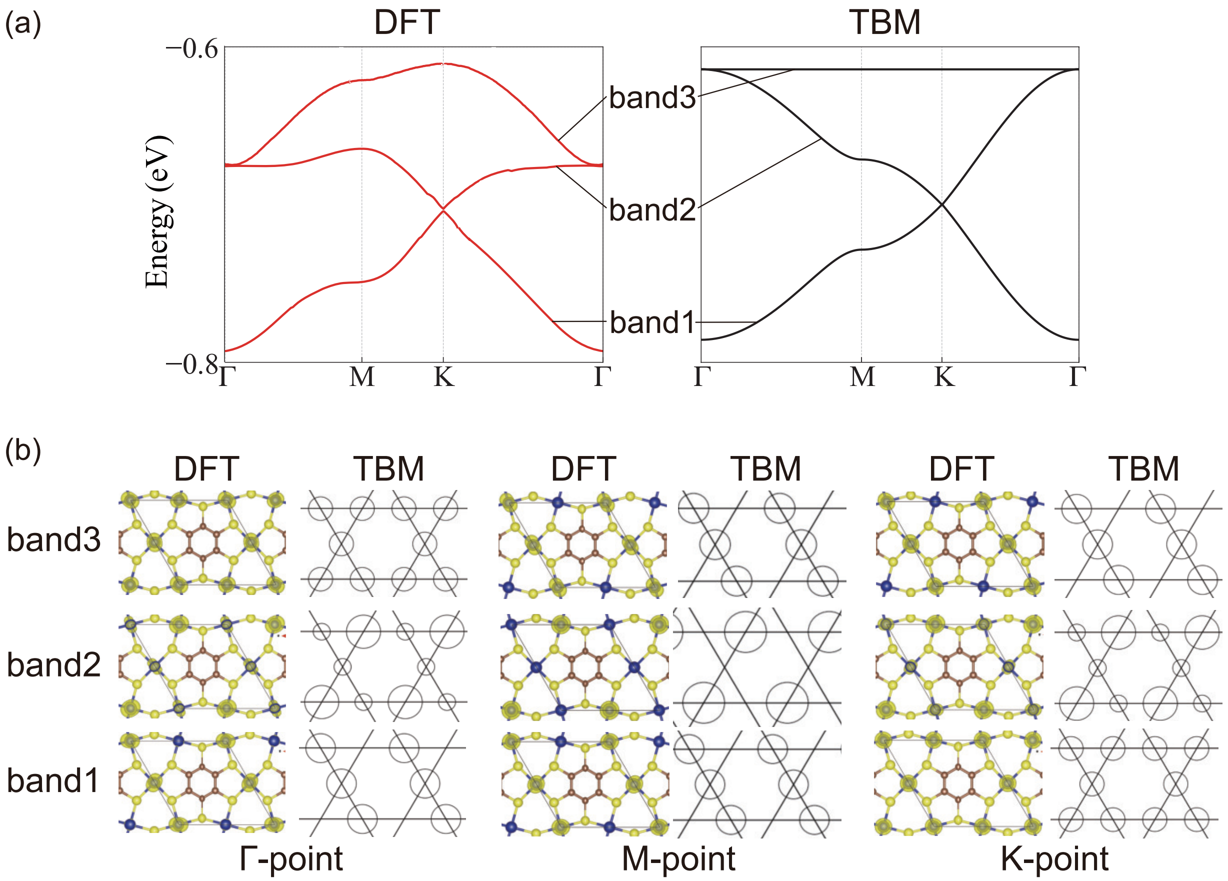

FIG. S2. (a) (left) Spin-up state energy subbands of CoBHT for HDS in the range of $-0.8 \leq E \leq -0.6$ eV. It is obtained from DFT calculation. (right) Energy band structure of 2D kagome lattice obtained from the nearest-neighbor tight-binding model. (b) Comparison of charge density profile between DFT and tight-binding model at Γ, K, M points.

at the site $\alpha(=A, B, C)$ for the n-th band. The Hamiltonian matrix can be written as

$$\mathcal{H}(\boldsymbol{k}) = -t \begin{pmatrix} 0 & 1+e^{-ik_1} & 1+e^{-ik_3} \\ 1+e^{ik_1} & 0 & 1+e^{-ik_2} \\ 1+e^{ik_3} & 1+e^{ik_2} & 0 \end{pmatrix} . \tag{S2}$$

Here, t represents the hopping energy between nearest-neighbor sites, and $k_\nu = \boldsymbol{k} \cdot \boldsymbol{a}_\nu (\nu = 1, 2, 3)$, where $\boldsymbol{a}_3 = \boldsymbol{a}_1 + \boldsymbol{a}_2$ is defined. Therefore, the eigenvalues of the kagome lattice are given by

$$E_{(1,2),\boldsymbol{k}} = -t \left\{ -1 \pm \sqrt{4A_{\boldsymbol{k}} - 3} \right\} , \tag{S3}$$

$$E_3 = -2t , \tag{S4}$$

where $A_{\boldsymbol{k}} = \cos^2 k_1 + \cos^2 k_2 + \cos^2 k_3$.

The left panel of Fig. S2(a) shows the energy band structure obtained by DFT calculation for HDS of CoBHT. These energy bands are only spin-up states subbands in the energy range of $-0.8 \leq E \leq -0.6$ eV. The whole band structure can be found in Fig. 2(c) in the main text. The right-panel of Fig. S2(a) shows the energy band structure obtained by the tight-binding model, where we have set $t = -1$ in Eq. (S4). We can clearly see the similarity between these two results. In both bands, bands 1 and 2 exhibit linear dispersion at the K point, and bands 2 and 3 connect at the Γ point. Regarding the DFT bands, band 3 is not completely flat owing to the interaction between Co and bis(dithiolene).

Figure S2(b) presents a comparison between the charge density obtained from DFT and that obtained from the tight-binding model. We obtained charge density for each of the three bands at the high symmetry points Γ, M, and K. The charge density is given by the square of the absolute value of the wave function. The distribution and magnitude of charge density are consistent for DFT and the tight-binding model, respectively. Therefore, the kagome-like band in HDS of CoBHT is attributed to the kagome structure formed by cobalt atoms.

II. ENERGY BAND STRUCTURE OF HDS UNDER UNIAXIAL STRAIN

Here, we show the energy band structure of CoBHT for HDS with uniaxial strain. In the main text, we have shown only the case of strong elongation strain for HDS over 1.0%. Here, we show the weaker strain case both for elongation and compression strain less than 1.0%. Figure S3 shows the energy band structure under elongation strain from 0 to 0.5%. Figure S4 shows the density of states for 0 and 0.5 elongation strain. Figure S5 shows the energy band structure under compression strain from 0 to 0.5%. It should be noted that we cannot observe the anisotropy of strain in these weak strain region.

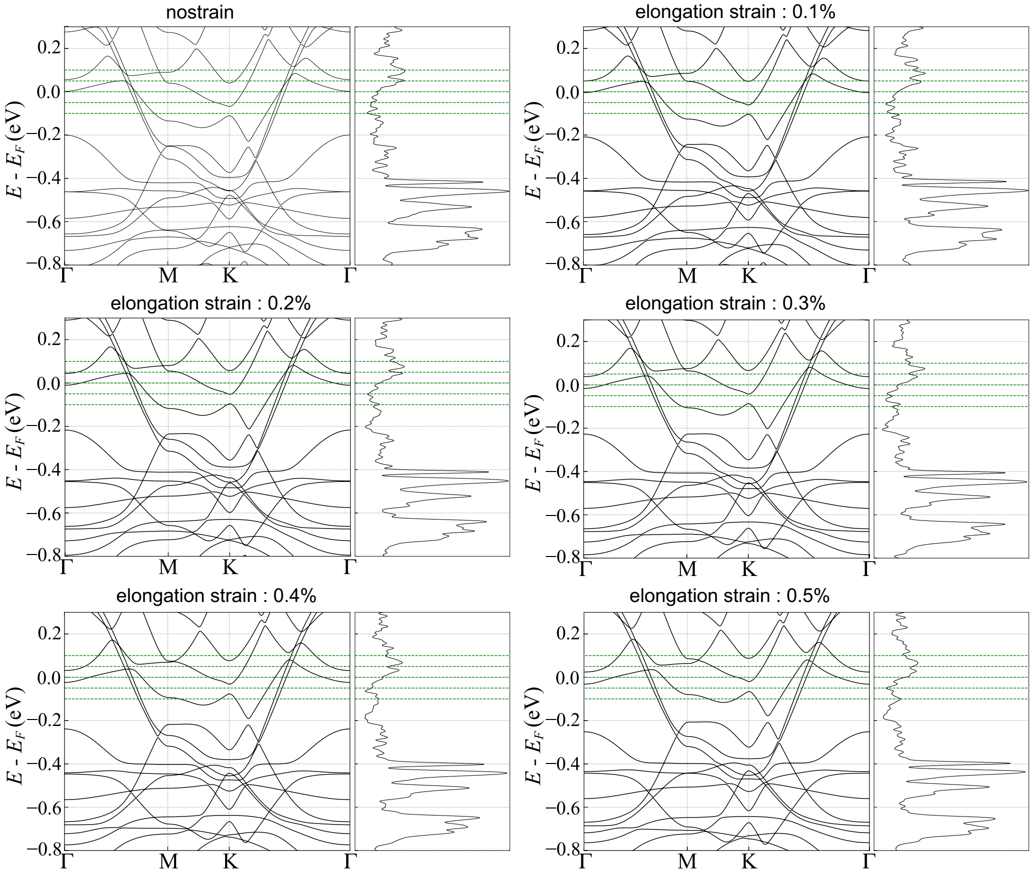

FIG. S3. Energy band structure of CoBHT for HDS under uniaxial elongation strain from 0 to 0.5%.

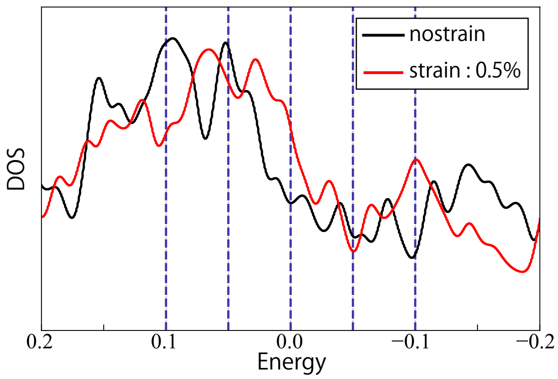

FIG. S4. Density of states of HDS for 0 and 0.5%.

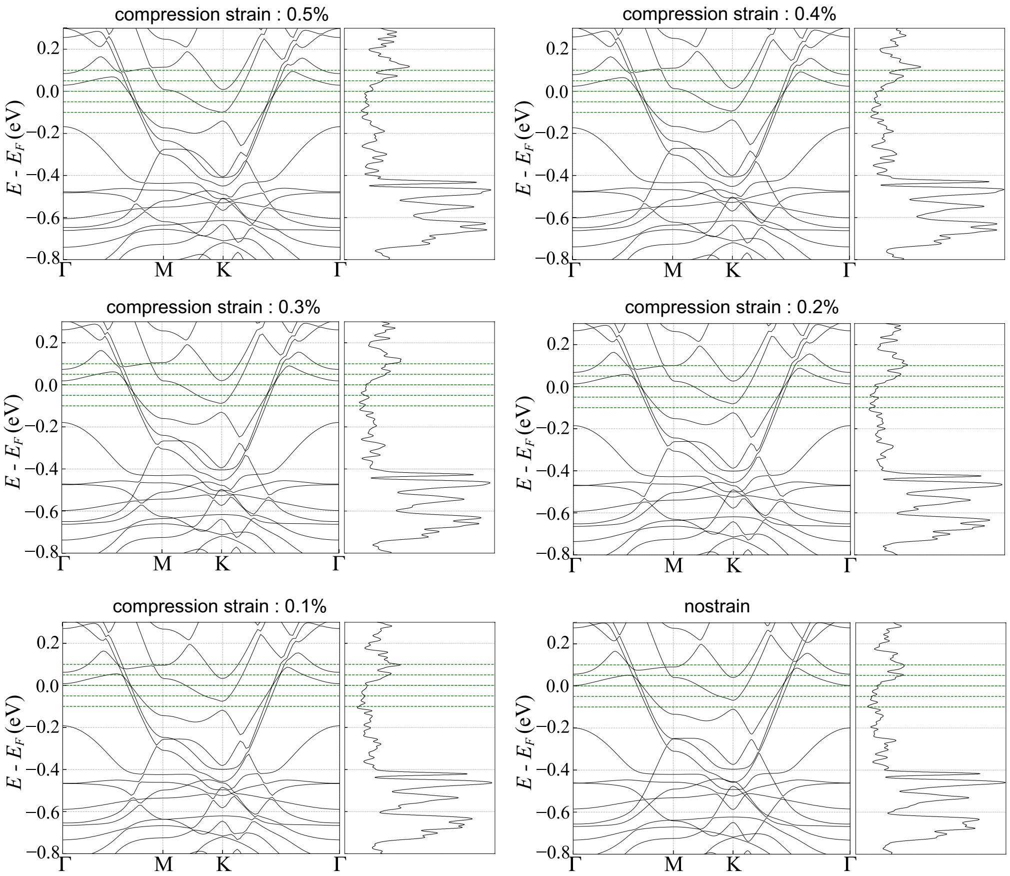

FIG. S5. Energy band structure of CoBHT for HDS under uniaxial compression strain from 0 to 0.5%.

III. ANISOTROPY IN ENERGY BAND STRUCTURES OF COBHT UNDER STRONG UNIAXIAL STRAIN

In the weak strain region up to about 0.5%, we cannot observe the anisotropic effect of uniaxial strain on the energy band structures of CoBHT. However, if the uniaxial strain goes beyond about 1.0%, the anisotropic effect of uniaxial strain on energy band structures appears. Here we show the calculation data for energy band structures of CoBHT both for HDS and LDS under strong uniaxial strain beyond 1.0%.

Figure S6 shows the energy band structures of HDS under strain. Upper panels show the compression strain from 1.0, 5.0, and 10.0% along x-axis. Similarly, lower panels show the energy band structures under the compression strain along y-axis.

Figure S7 shows the energy band structures of LDS under strain. Upper panels show the elongation strain from 1.0, 5.0, and 10.0% along x-axis. Similarly, lower panels show the energy band structures under the elongation strain along y-axis.

Figure S8 shows the energy band structures of LDS under strain. Upper panels show the compression strain from 1.0, 5.0, and 10.0% along x-axis. Similarly, lower panels show the energy band structures under the compression strain along y-axis.

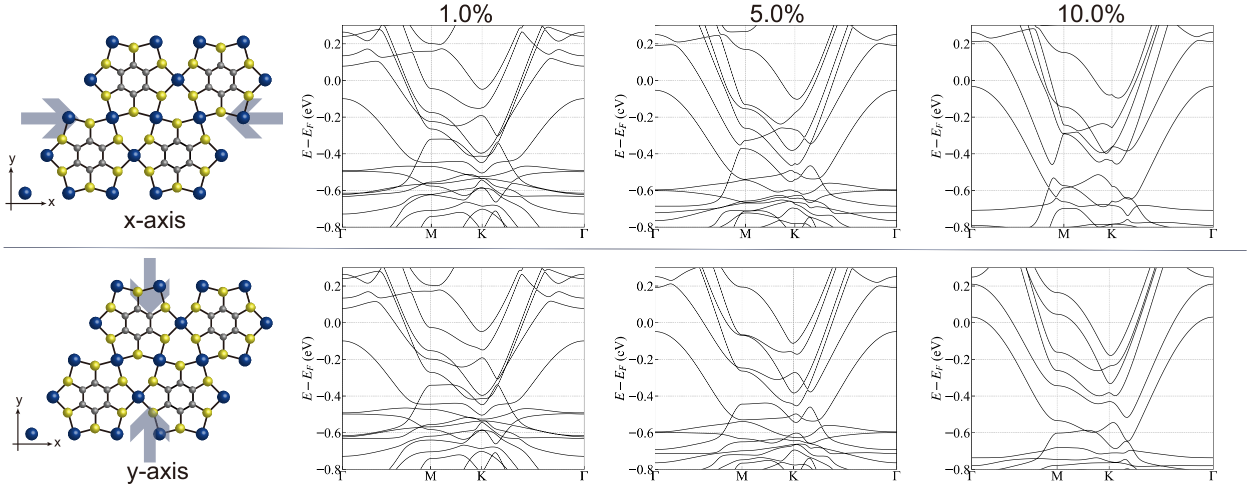

FIG. S6. Energy band structures of HDS under strain. Upper panels show the compression strain from 1.0, 5.0, and 10.0% along x-axis. Similarly, lower panels show the energy band structures under the compression strain along y-axis.

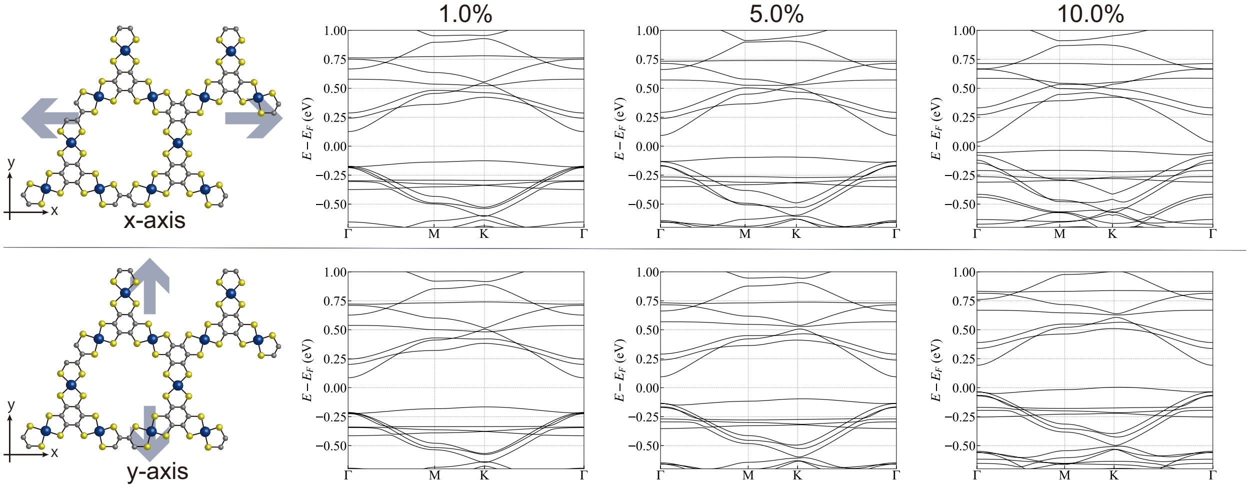

FIG. S7. Energy band structures of LDS under strain. Upper panels show the elongation strain from 1.0, 5.0, and 10.0% along x-axis. Similarly, lower panels show the energy band structures under the elongation strain along y-axis.

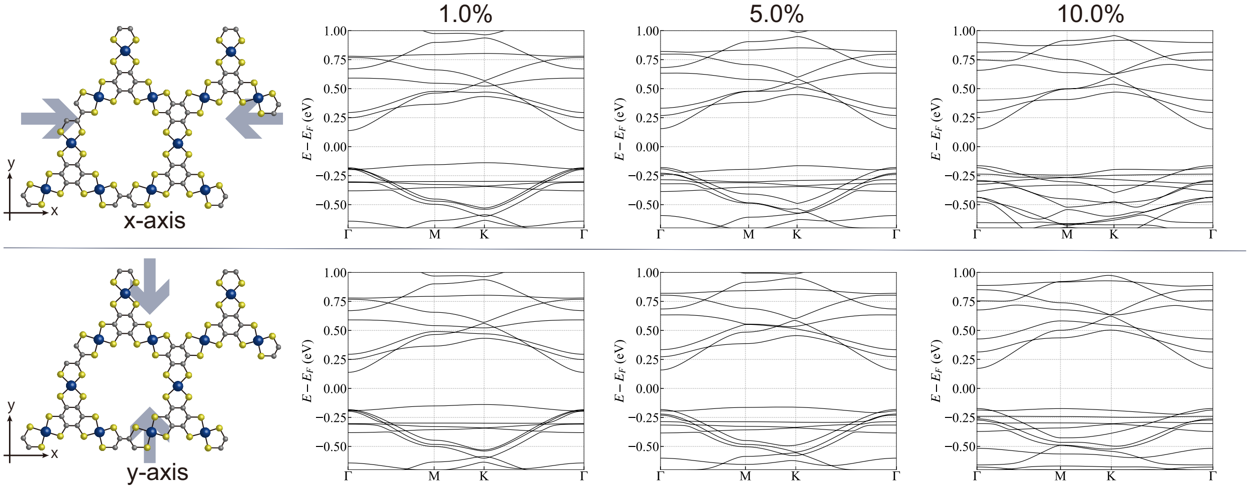

FIG. S8. Energy band structures of LDS under strain. Upper panels show the compression strain from 1.0, 5.0, and 10.0% along x-axis. Similarly, lower panels show the energy band structures under the compression strain along y-axis.

IV. MOLECULAR DYNAMICS RESULTS OF COBHT AT 300 K

We verify the thermal stability of CoBHT using ab initio molecular dynamics (AIMD) simulations in the NVT ensemble at 300 K, as implemented in VASP. A 2 × 3 supercell is considered for the HDS phase (see FIG. S9) and a 2 × 2 supercell for the LDS phase (see FIG. S10). Using the Andersen thermostat, the simulations are run for 2000 steps (i.e., 1 ps) with a time step of 0.5 fs. From the top and side views, as well as the total structure of both phases after 2000 steps (at 300 K), their hexagonal configurations and planarity remain intact, which strongly confirms their thermal stability.

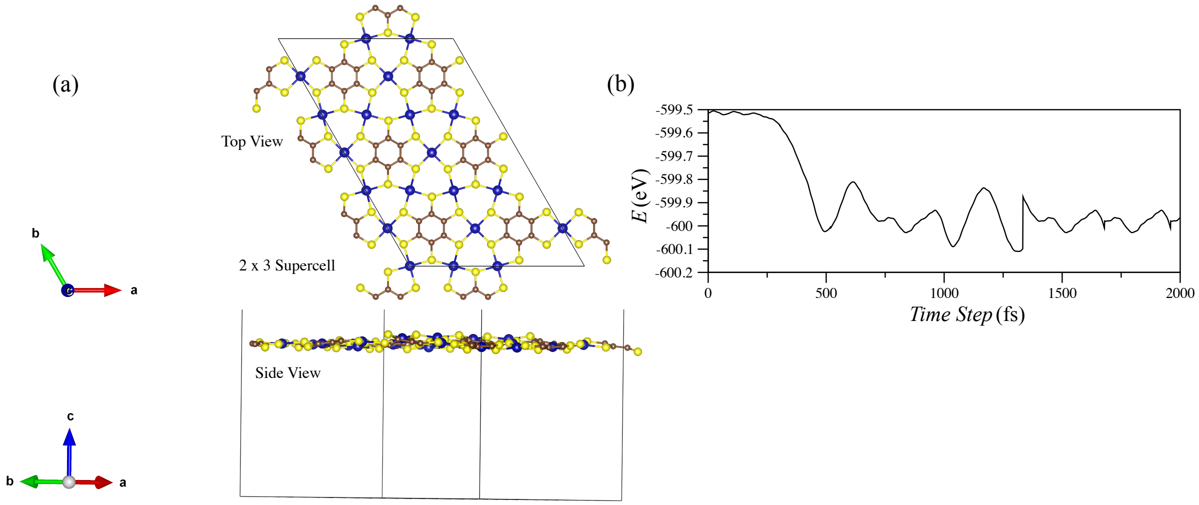

FIG. S9. The (a) top view and side view of CoBHT in HDS phase after 2000 AIMD steps with 2×3 supercell. (b) Total energy variation of CoBHT in HDS phase with respect to time step length of 0.5 fs.

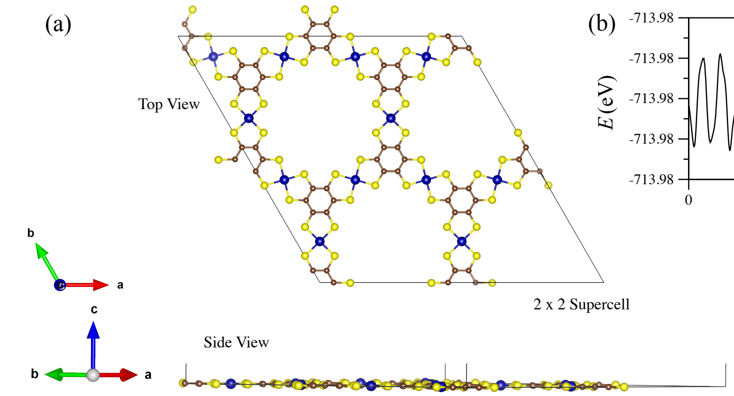

FIG. S10. The (a) top view and side view of CoBHT in LDS phase after 2000 AIMD steps with 2×2 supercell. (b) Total energy variation of CoBHT in LDS phase with respect to time step length of 0.5 fs.

V. ELECTRONIC BAND STRUCTURES OF COBHT USING DFT+U METHOD

We vary U from U = 0 to U = 3 eV on each Co atoms. We plot electronic band structure at different U in FIG. S11 and FIG.S12 in HDS and LDS phase respectively. In the case of the HDS phase, we observe that the positions of the bands near the Fermi level remain the same at different values of U. However, far below the Fermi level, the band positions change slightly, which does not affect the magnetic ground state. In addition, we find that the magnetic moment on each Co atom remains the same as at U = 0 eV compared to other U values. Therefore, CoBHT in the HDS phase exhibits a ferromagnetic ground state. We find similar results in the case of the LDS phase (see FIG. S12). In conclusion, the on-site Coulomb potential does not affect their magnetic ground state.

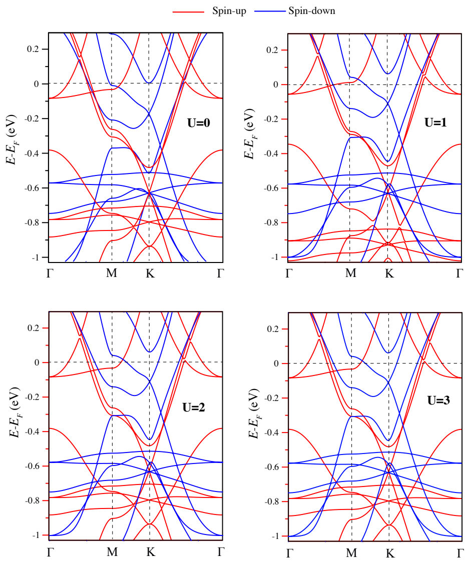

FIG. S11. Electronic band structures of CoBHT in HDS phase with different U (on site Coulomb energy on each Co atoms). The horizontal dashed lines present the Fermi level.

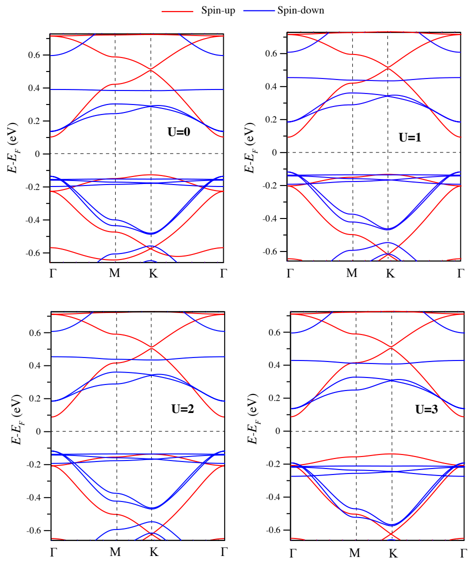

FIG. S12. Electronic band structures of CoBHT in LDS phase with different U (on site Coulomb energy on each Co atoms). The horizontal dashed lines present the Fermi level.
